# Molecular and Functional Cargo of Plasma-Derived Exosomes in Patients with Hereditary Hemorrhagic Telangiectasia

**DOI:** 10.3390/jcm13185430

**Published:** 2024-09-13

**Authors:** Yanru Wang, Linda Hofmann, Diana Huber, Robin Lochbaum, Sonja Ludwig, Cornelia Brunner, Thomas K. Hoffmann, René Lehner, Marie-Nicole Theodoraki

**Affiliations:** 1 Department of Otorhinolaryngology, Head and Neck Surgery, Ulm University Medical Center, 89075 Ulm, Baden-Wuerttemberg, Germany; 2 Department of Otorhinolaryngology, Head and Neck Surgery, University Hospital Mannheim, Medical Faculty Mannheim, University of Heidelberg, 68167 Mannheim, Baden-Wuerttemberg, Germany; 3 Core Facility Immune Monitoring, Ulm University Medical Faculty, 89075 Ulm, Baden-Wuerttemberg, Germany; 4 Department of Otorhinolaryngology, Head and Neck Surgery, Klinikum Rechts der Isar, Technical University Munich, 81675 Munich, Bavaria, Germany

**Keywords:** hereditary hemorrhagic telangiectasia (HHT), exosome, biomarker, thrombospondin-1 (THBS1), soluble endoglin (sENG), endoglin (ENG), ALK1, ALK5

## Abstract

**Background**: Hereditary Hemorrhagic Telangiectasia (HHT) is a genetic disorder leading to frequent bleeding in several organs. As HHT diagnosis is demanding and depends on clinical criteria, liquid biopsy would be beneficial. Exosomes from biofluids are nano-sized vesicles for intercellular communication. Their cargo and characteristics represent biomarkers for many diseases. Here, exosomes of HHT patients were examined regarding their biosignature. **Methods**: Exosomes were isolated from the plasma of 20 HHT patients and 17 healthy donors (HDs). The total exosomal protein was quantified, and specific proteins were analyzed using Western blot and antibody arrays. Human umbilical vein endothelial cells (HUVECs) co-incubated with exosomes were functionally examined via immunofluorescence, proliferation, and scratch assay. **Results**: The levels of the angiogenesis-regulating protein Thrombospondin-1 were significantly higher in HHT compared to HD exosomes. Among HHT, but not HD exosomes, a negative correlation between total exosomal protein and soluble Endoglin (sENG) levels was found. Other exosomal proteins (ALK1, ALK5) and the particle concentration significantly correlated with disease severity parameters (total consultations/interventions, epistaxis severity score) in HHT patients. Functionally, HUVECs were able to internalize both HD and HHT exosomes, inducing a similar change in the F-Actin structure and a reduction in migration and proliferation. **Conclusions**: This study provided first insights into the protein cargo and function of HHT-derived exosomes. The data indicate changes in sENG secretion via exosomes and reveal exosomal Thrombospondin-1 as a potential biomarker for HHT. Several exosomal characteristics were pointed out as potential liquid biomarkers for disease severity, revealing a possible new way of diagnosis and prognosis of HHT.

## 1. Introduction

Hereditary Hemorrhagic Telangiectasia (HHT, Osler-Weber-Rendu syndrome) is a rare genetic disorder with a prevalence of 1 in 5000–8000 people, which leads to the instability of blood vessels [1]. Patients who suffer from HHT often experience spontaneous nose bleeding and noticeable telangiectasia on skin and mucosal tissue [2]. The most common symptom is epistaxis, which recurrently and severely affects more than 90% of patients [2,3]. Iron deficiency anemia is a well-known side effect, which can be intensified by gastrointestinal telangiectasia and further visceral arterio-venous malformations (AVM) [4]. Pulmonary, hepatic, and cerebral AVMs can occur with different frequencies. They are most likely to remain asymptomatic but can also be fatal [5,6,7]. Therapeutic measures are limited to mainly AVM management and symptomatic care. Even though different systemic and topical treatment regimens are being conducted, for example, anti-angiogenic therapies with bevacizumab, there is no targeted treatment available yet [8,9].

HHT is an autosomal dominant disorder caused by different mutations. The two most commonly mutated proteins are Endoglin (ENG) and activin receptor-like kinase (ACVRL1 or ALK1) [1,2], both being part of the transforming growth factor beta (TGF-ß) signaling pathway in endothelial cells. This signaling pathway is highly dysfunctional in HHT patients. The resulting endothelial malfunction [10,11,12], especially the impaired angiogenesis, leads to arteriovenous malformations, resulting in the above-mentioned typical clinical symptoms such as nose bleeding [1].

While the transmembrane form of ENG is essential for angiogenesis in endothelial cells, its extracellular part can be proteolytically separated and released into the blood [13]. This circulating protein is called soluble Endoglin (sENG). sENG is involved in the pathogenesis of several endothelium-related diseases, such as preeclampsia [14], and, additionally, modulates angiogenesis and other endothelial functions such as migration, which can reduce vascular abnormalities found in HHT mouse models [15,16]. However, the effects of sENG in HHT pathophysiology are not fully understood yet.

According to the current international guidelines, HHT diagnosis is mainly based on clinical criteria called the “Curaçao criteria” [2]. Genetic testing is primarily used to identify suspected HHT mutations in relatives of HHT patients to confirm diagnosis or for research purposes [2]. However, the sensitivity of genetic testing is only estimated to be between 70 and 85% [2]. Thus, more reliable, objective, and simple diagnostic methods such as liquid biomarkers are needed to improve the diagnostic progress and accuracy.

Once the diagnosis is confirmed, there are few reliable ways to monitor the disease progression. The Epistaxis Severity Score (ESS) [17] helps to evaluate the current disease severity. Body imaging can detect new AVMs as signs of disease progression. However, earlier detection of disease activity is desirable as future targeted therapies could prevent the formation of new AVMs. Thus, liquid biomarkers are needed for better disease monitoring.

Exosomes are nano-sized extracellular vesicles (sEVs) carrying proteins and genetic material resembling the characteristics of their parental cells [18]. Exosomes can move freely in all body fluids and are important tools for intercellular communication upon internalization or binding to cell membrane proteins [19,20,21,22]. Recent research identified exosomes as potential liquid biomarkers not only for several types of cancer, including head and neck or breast cancer [23,24,25], but also for neurodegenerative [26,27] and cardiovascular diseases [28,29]. Various components of the exosome cargo and characteristics have the potential to serve as disease biomarker, such as proteins [23], RNA [26,30], DNA [31], lipids [32], and spectral properties [25]. So far, there has been one study investigating the potential of plasma-derived exosomes as biomarkers for HHT by evaluating their microRNA cargo [33], but their protein cargo and functions still remain unclear. 

This study provides a characterization of plasma-derived exosomes from HHT patients and presents one of the biggest HHT cohorts reported so far. Differences between exosomes from healthy donors (HDs) and HHT patients and the differential exosomal effect on endothelial cells are examined to find new potential liquid biomarkers for HHT and reveal new targets for future HHT pathophysiology research. 

## 2. Materials and Methods

### 2.1. Patients and Plasma Samples

This study was retrospectively conducted according to the Declaration of Helsinki and approved by the local ethics committee of Ulm University (#108/20). A total of 20 HHT patients and 17 age- and gender-matched HDs gave informed consent and donated blood at the Department of Otorhinolaryngology, Ulm University Medical Center from 2020 to 2022. Sodium citrate blood was centrifuged at 1000× *g* for 10 min followed by 2500× *g* for 10 min. Plasma samples were stored at −20 °C.

### 2.2. Exosome Isolation by Size Exclusion Chromatography (SEC)

Exosomes were isolated from plasma samples via size exclusion chromatography (SEC), as previously described [34]. First, plasma samples were centrifuged at 2000× *g* for 10 min and 12,000× *g* for 30 min at 4 °C. Then, 0.22 μm syringe filters (Millipore, Burlington, MA, USA, SLGPO33RB) were used for supernatant filtration. An amount of 1 mL of the resulting plasma was loaded on 10 mL sepharose SEC columns (Bio-Rad, Hercules, CA, USA, 732–1011) and eluted with PBS. The exosome-enriched fraction #4 [34] was collected. Isolated exosomes were stored at 4 °C and used within 1 week. Exosomes for Western blot were frozen at −20 °C for later use.

### 2.3. BCA and Exosome Characterization

The Pierce BCA Protein Assay (ThermoFisher Scientific, Waltham, MA, USA, 23225) was performed to quantify total exosomal protein (TEP) concentration according to the manufacturer’s protocol. Amicon Ultra Centrifugal Filter Units (Millipore, UFC5100BK) were used to concentrate exosomes to desired volumes. For Western blot, 16–20 µg of exosomes in 30 µL PBS were used; for functional assays, 10 µg of exosomes in 100 µL were used; for antibody arrays, 100 µg of exosomes in 1 mL were used.

Following the minimal information for studies of extracellular vesicles (MISEV) 2018 guidelines for the definition of EVs [35], exosomes were characterized via transmission electron microscopy (TEM), nanoparticle tracking analysis (NTA), and Western blot, as previously described [23] (EV-TRACK ID: EV200068). Protein per particle was calculated as the ratio of TEP (as determined by BCA) and particle number (as determined by NTA).

### 2.4. Western Blot Analysis of Exosomes

Exosomes were lysed with (non-)reducing buffer (Thermo Fisher, Waltham, MA, USA, 39000 & 39001). Proteins were separated on polyacrylamide gels (Bio-Rad, 456–1044/456–1094) and transferred to PVDF or nitrocellulose membranes. Antibodies used are shown in Table 1. Signal intensities of sENG bands detected by the ChemiDoc MP Imaging System (Bio-Rad) were normalized to TSG101 using lane normalization factors.

### 2.5. Angiogenesis Array

The Proteome Profiler Human Array Kit (Bio-Techne, Minneapolis, MN, USA, ARY007) was performed according to the manufacturer’s protocol. Signals were detected using the ChemiDoc MP Imaging System (Bio-Rad).

### 2.6. Cell Culture

Cryopreserved HUVECs (human umbilical vein endothelial cells) from pooled donors (PromoCell, Heidelberg, Germany, C-12203) were cultured in Endothelial Cell Growth Medium 2 Kit (PromoCell, C-22111) and split using DetachKit (C-41220, all from PromoCell). Passages 2 to 5 and exosome-depleted FBS (fetal bovine serum) (Gibco, Waltham, MA, USA, A2720801) were used for functional assays.

### 2.7. Exosome Internalization and F-Actin Assay

Exosomes were labeled with 5 µL of PKH26 cell linker diluted in Diluent C (Sigma-Aldrich, St. Louis, MO, USA, MIDI26-1KT). HUVECs were incubated with labeled exosomes for 1 h, 4 h, or 16 h. HUVECs were incubated with PBS, HD or HHT exosomes for 24 h. Cells were fixed using 4% paraformaldehyde (Thermo Scientific, Waltham, MA, USA, 28906), permeabilized using 0.5% Triton X (Sigma-Aldrich, T8787-50ML) and stained with F-Actin (Invitrogen, Waltham, MA, USA, R37110) and DAPI (Invitrogen, P36935). Results were viewed under a light microscope.

### 2.8. Tube Formation Assay

HUVECs were seeded to plates containing extracellular matrix (Abcam, Cambridge, UK, ab204726) and directly incubated with PBS as control, HD, or HHT exosomes for 16 h. Cells were stained according to the manufacturer’s protocol and documented using a light microscope. The total length of tubes was calculated using ImageJ 1.53k macro [36].

### 2.9. Proliferation Assay

HUVECs were labeled using CFSE (Abcam, ab113853) according to the manufacturer’s protocol and incubated with PBS, HD or HHT exosomes for 24 h. Fluorescence intensity of control and exosome-primed HUVECs was measured via flow cytometry. For quantification, percentages of proliferated cells were calculated.

### 2.10. Wound Healing Assay

HUVECs were incubated with PBS, HD, or HHT exosomes for 24 h. Scratches were created using 200 µL pipette tips and documented under a light microscope at 0 h, 24 h, and 48 h after scratch induction. Scratch gaps were calculated using ImageJ 1.53k macro [37].

### 2.11. Statistics

GraphPad Prism 10.3.1 (San Diego, CA, USA) was used for statistical analysis and visualization. Independent samples were compared using Mann–Whitney test. Wilcoxon signed-rank test was used for paired samples. Spearman’s rank correlation test was performed on non-normally distributed data, and Pearson correlation test was performed on normally distributed data.

## 3. Results

### 3.1. Study Population

The characteristics of the included cohort of HHT patients and age- and gender-matched HDs are listed in Table 2. The diagnosis of HHT was based on Curaçao criteria, and data from genetic testing were available for two patients (Table S1). At the time point of the blood draw, the mean age of HHT patients was 57 years with a range from 22 to 87 years. A total of 55% of patients were female while 45% were male. The most common location of disease manifestation was in the nose (95%), oral cavity (60%), and lips (45%). A total of 50% of the patients had 4–6 points in their initial ESS at the time of study inclusion. A total of 35% and 65% of patients received less than 10 total consultations and total interventions, respectively. Detailed data on the interventions for individual patients are shown in Table S2.

### 3.2. Exosome Characterization

**Figure 1:**
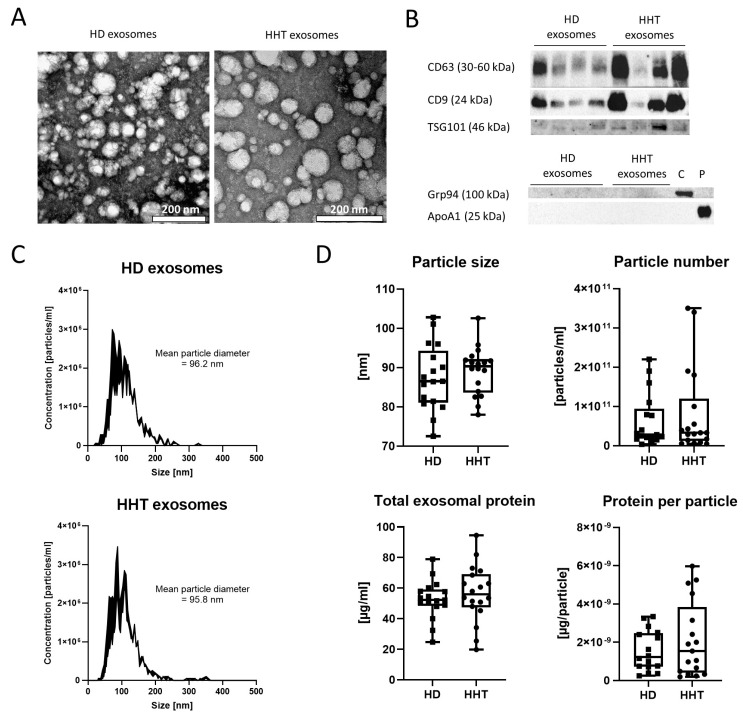
Exosome characterization. (**A**) Representative transmission electron microscopy images of plasma-derived exosomes from a healthy donor (HD) and a patient with Hereditary Hemorrhagic Telangiectasia (HHT). Scale bar = 200 nm. (**B**) Western blot analysis of HD and HHT exosomes for exosomal markers (CD63, CD9, TSG101), the cellular marker Grp94, and lipoprotein ApoA1. A cell lysate (C) and unprocessed plasma (P) served as positive controls. (**C**) Representative size distributions of plasma-derived exosomes from HD and HHT patient determined via nanoparticle tracking analysis. (**D**) Quantitative characteristics of plasma-derived exosomes from HD (n = 17) and HHT patients (n = 18). Box-and-whisker plots represent the median, the 25th and 75th quartiles, and the range.

Exosomes isolated from plasma of HDs and HHT patients were characterized following the MISEV 2018 guidelines [35]. TEM confirmed the presence of intact vesicles in exosome preparations (Figure 1A). Most vesicles had a diameter of less than 100 nm. They appeared to be round-shaped with electron-dense outer layers—a reflection of proteins anchored in the vesicle membrane—and less electron-dense inner bodies.

Moreover, Western blot confirmed the presence of exosomal markers CD63, CD9, and TSG101 in exosome preparations from both HDs and HHT patients (Figure 1B). While Grp94 was detected in a cell lysate and ApoA1 in a plasma sample, their absence was confirmed in exosome preparations (Figure 1B). Representative size distribution histograms for one matched HD/HHT pair showed particles <200 nm with a mean diameter of approximately 95 nm (Figure 1C). No significant differences were found in mean particle size (*p* = 0.42), particle number (*p* = 0.91), total exosomal protein (TEP, *p* = 0.40), and protein per particle (*p* = 0.90) between HD and HHT exosomes (Figure 1D).

### 3.3. Soluble Endoglin Levels in HD and HHT Exosomes

**Figure 2:**
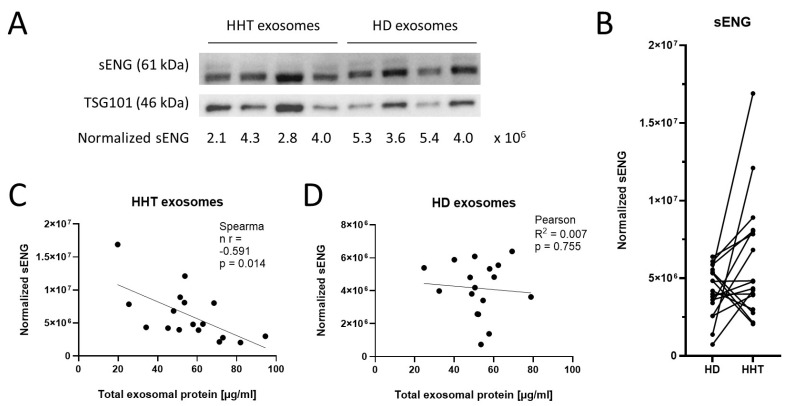
Soluble Endoglin levels in HD and HHT exosomes. (**A**) Representative Western blot of HD and HHT exosomes for the angiogenesis-related protein sENG and the exosomal marker TSG101. Numbers below lanes indicate band intensities of sENG normalized to TSG101 using lane normalization factors. (**B**) Normalized sENG values of HD and HHT exosomes (n = 17). Age- and gender-matched pairs of HDs and HHT patients whose plasma was used for exosome isolation are connected by a line. (**C**) Normalized sENG values of HHT exosomes (n = 17) were correlated to total exosomal protein levels, as determined by Bicinchoninic acid (BCA) assay. Spearman’s rank correlation coefficient (r) and correlation significance (p) were calculated. (**D**) Normalized sENG values of HD exosomes (n = 17) were correlated to the total exosomal protein levels, as determined by BCA assay. Pearson coefficient of correlation (R2) and correlation significance (p) are shown.

Soluble Endoglin (sENG) and the exosomal protein TSG101 were analyzed in HD and HHT exosomes (Figure 2A). Although no clear significance could be determined among the normalized sENG values of HD and HHT exosomes (*p* = 0.057), these data showed a clear trend of higher sENG levels in HHT patients compared to matched HDs (Figure 2B). A significant negative correlation between the level of sENG and TEP was found in HHT patients (Figure 2C, *p* = 0.014) but not HDs (Figure 2D).

### 3.4. Angiogenesis-Related Proteins in HD and HHT Exosomes

**Figure 3:**
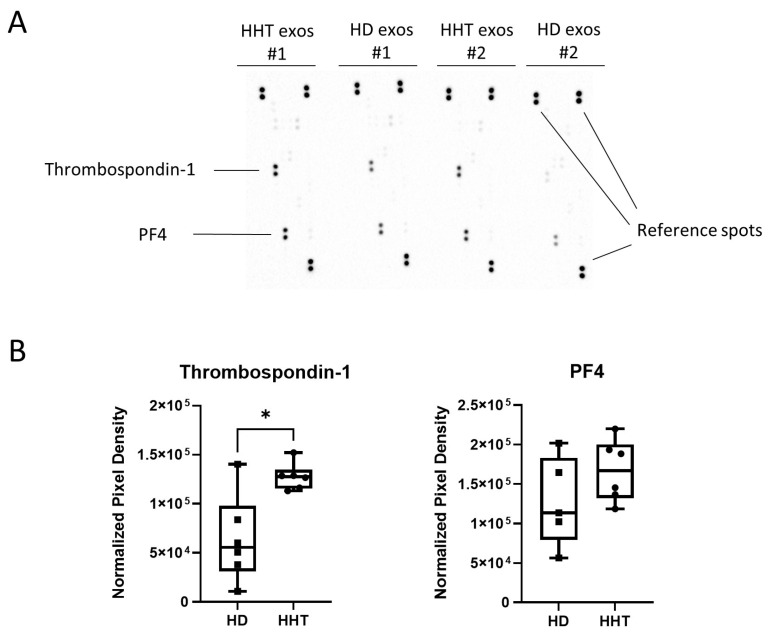
Angiogenesis-related proteins in HD and HHT exosomes. (**A**) Representative antibody array analysis of HD and HHT exosomes (n = 2) for angiogenesis-related proteins including Thrombospondin-1 and platelet factor 4 (PF4). The reference spots’ signal density was used for normalization and quantification of protein levels. (**B**) Normalized pixel density of Thrombospondin-1 and PF4 was compared between HD and HHT exosomes (n = 6). Box-and-whisker plots represent the median, the 25th and 75th quartiles, and the range. Mann–Whitney test was applied for comparison between HD and HHT exosomes with * corresponding to *p* ≤ 0.05.

Other angiogenesis-related proteins were analyzed with Proteome Profiler Human Angiogenesis Arrays. Thrombospondin-1 and Platelet factor 4 (PF4) were detected in HD and HHT exosomes (Figure 3A). The exosomal levels of Thrombospondin-1 were significantly higher in HHT patients compared to HDs (Figure 3B, *p* = 0.041). A trend towards higher exosomal levels of PF4 was also visible in HHT patients (Figure 3B).

### 3.5. Structural Changes in HUVECs after Exosome Incubation

**Figure 4:**
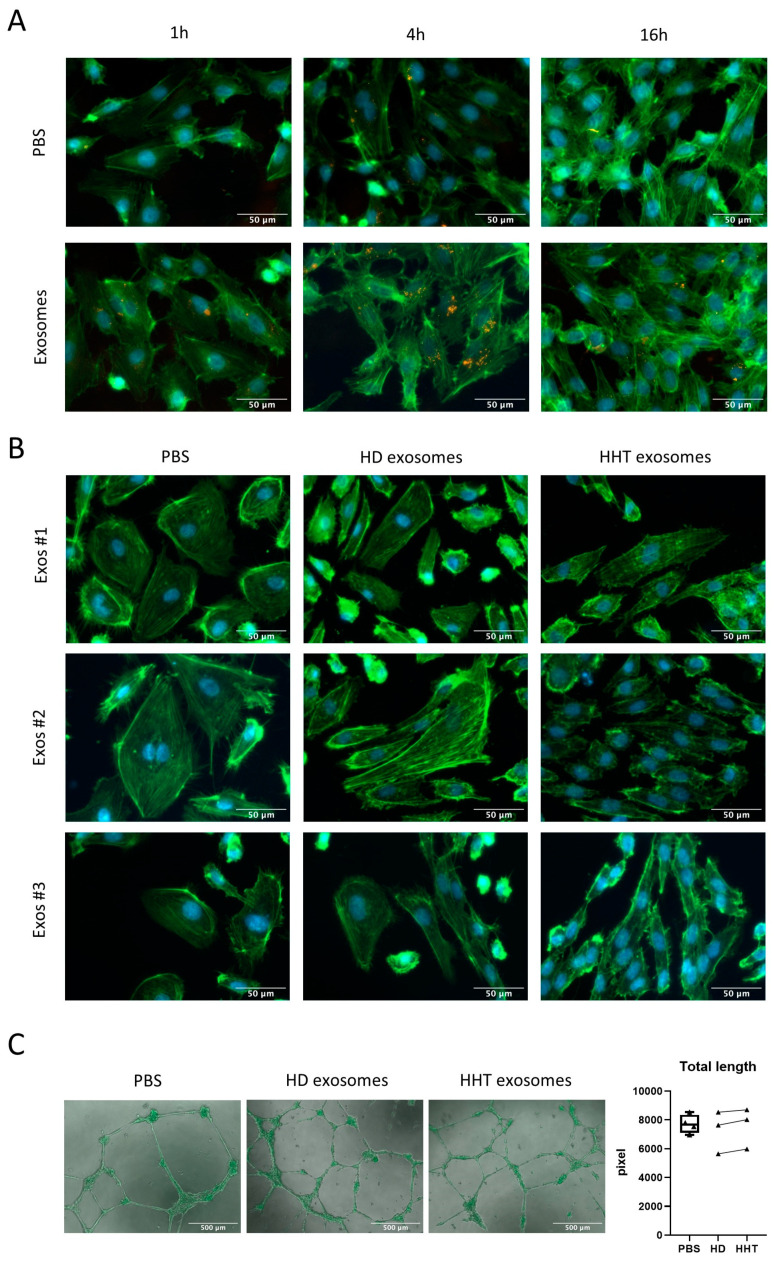
Structural changes in HUVECs after exosome incubation. (**A**) Fluorescence microscopy images at 400× magnification, scale bar = 50 µm. HUVECs were incubated with PKH26-labeled exosomes (orange) for 1 h, 4 h, or 16 h. F-Actin filaments are shown in green, and nuclei in blue (DAPI). (**B**) Representative images of F-actin structure in HUVECs after exosome incubation. HUVECs were incubated with PBS as control, HD exosomes, or HHT exosomes for 24 h. F-Actin filaments are shown in green, and nuclei in blue (DAPI). (**C**) Tube formation assay on HUVECs after exosome incubation. HUVECs grown in an extracellular matrix were incubated with PBS as control (n = 4), HD, or HHT exosomes (n = 3) for 16 h. Representative fluorescence microscopy images (50× magnification, scale bar = 500 µm) show the results of tube formation. Using ImageJ 1.53k, the total length of tubes in the analyzed area was calculated for the three groups of treated HUVECs.

Previous studies have shown abnormalities of HHT endothelial cells regarding their cell shape, F-actin organization, and tube formation ability in comparison to control endothelial cells [10,11]. Thus, the effect of plasma-derived HHT exosomes on the disruption of healthy endothelial cells as a possible driver of HHT pathogenesis was examined. To determine if exosomes can convey cargo and function to HUVECs at all, uptake assays were performed. HUVECs were able to internalize exosomes with the highest rate after 4 h of co-incubation (Figure 4A).

To assess structural changes in the cytoskeleton, F-Actin filaments were stained after incubating HUVECs with PBS as control or plasma-derived exosomes (Figure 4B). As expected, PBS-treated HUVECs showed an organized cytoskeleton structure and dense and parallelly oriented F-actin filaments. After incubation with either HD or HHT exosomes, the filaments seemed disorganized and less dense. Additionally, many dots of high-intensity, green F-actin fluorescence were detected in exosome-incubated HUVECs, whereas they were barely present in PBS-treated HUVECs.

To investigate modulations in angiogenesis ability, tube formation assays were performed. The tube formation ability was not changed between PBS- and exosome-treated HUVECs (Figure 4C). Independent of the treatment condition, all tubes appeared to be robust and normal, with no difference in total length.

### 3.6. Proliferative and Migratory Ability of HUVECs after Exosome Incubation

**Figure 5:**
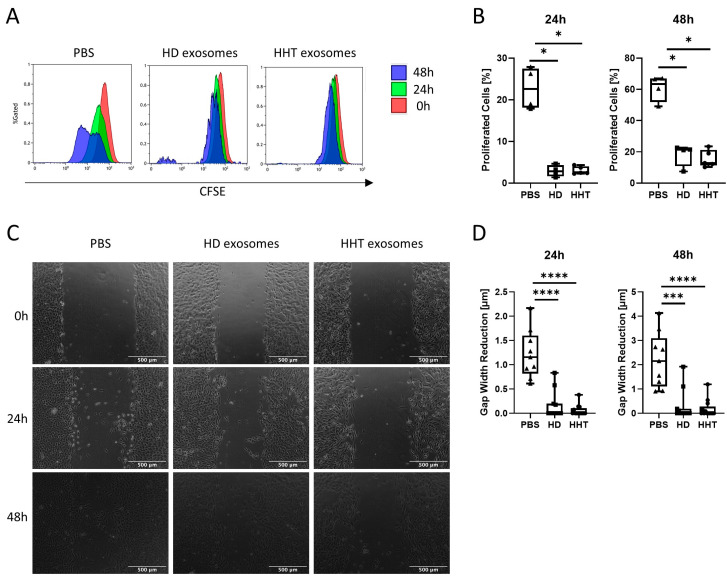
Proliferation and migration of HUVECs after exosome incubation. (**A**) Carboxy-fluorescein succinimidyl ester (CFSE) proliferation assay as determined via flow cytometry. CFSE-labeled HUVECs were incubated with PBS as control (n = 4), HD (n = 4) or HHT exosomes (n = 5) for 24 h. Then, these control and exosome-primed HUVECs were analyzed via flow cytometry after 0 h, 24 h, and 48 h. Representative flow cytometry histograms are shown. (**B**) Box-and-whisker blots show the median, the 25th and 75th quartiles, and the range of the percentage of proliferated HUVECs after 24 h and 48 h. Mann–Whitney test was applied for comparison between groups with * corresponding to *p* ≤ 0.05. (**C**) Wound healing assay of HUVECs after exosome incubation. Representative light microscopy images (50× magnification, scale bar = 500 µm) of HUVECs incubated with PBS as control (n = 9), HD (n = 11) or HHT exosomes (n = 13) for 24 h. Then, scratches were induced and documented 0 h, 24 h, and 48 h afterwards. (**D**) Using ImageJ, the gap width of each scratch was calculated (Figure S1). Box-and-whisker blots show the median, the 25th and 75th quartiles, and the range of the gap width reduction 24h and 48h after incubation. Mann–Whitney test was applied for comparison between groups with *** and **** corresponding to *p* ≤ 0.001 and *p* ≤ 0.0001.

The proliferative ability of HUVECs after incubation with PBS as control, HD, or HHT exosomes was assessed via CFSE assay and flow cytometry. Compared to the PBS control, exosome-primed HUVECS showed significantly reduced proliferation after 24 h and 48 h (Figure 5A, *p* < 0.05), with no difference between HD and HHT exosomes (Figure 5B). Changes in migratory ability were analyzed by wound healing assay (Figure 5C and Figure S1). Compared to the PBS control, exosome-primed HUVECS showed reduced migration at 24 h and 48 h after scratch induction (Figure 5D, *p* < 0.0001 and *p* < 0.001).

### 3.7. Correlations between Exosomal and Clinical Parameters

**Figure 6:**
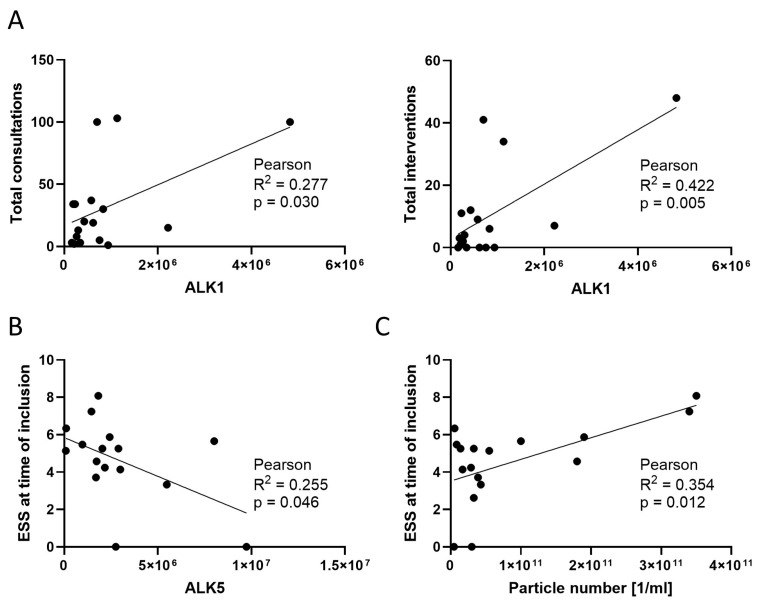
Correlations between exosomal and clinical parameters. (**A**) Normalized ALK1 values of HHT exosomes (n = 17) were correlated to the number of total consultations and total interventions. (**B**) Normalized ALK5 values of HHT exosomes (n = 16) were correlated to ESSs at the time of inclusion. (**C**) Particle concentration of HHT exosome samples (n = 17) was correlated to Epistaxis severity scores (ESS) at the time of inclusion. Pearson coefficient of correlation (R2) and correlation significance (p) are shown for all correlations.

To evaluate if exosomal characteristics resemble disease severity, all measured exosomal parameters were associated with clinical data from Table 2. Significant positive correlations between exosomal ALK1 and the number of total consultations (Figure 6A, *p* = 0.030) and the number of total interventions (Figure 6A, *p* = 0.005) were found in HHT patients. A significant negative correlation between the exosomal level of ALK5 and the initial ESS at the time point of study inclusion was found (Figure 6B, *p* = 0.046). Additionally, a significant positive correlation between the exosomal particle concentration and the ESS was observed (Figure 6C, *p* = 0.012).

## 4. Discussion

This is the first report characterizing exosomes isolated from the plasma of HHT patients and assessing differences between HD- and HHT-derived exosomes regarding their protein cargo and their impact on endothelial cells in vitro.

HD and HHT exosomes were found to be similar regarding particle size, number, and TEP. However, HHT exosomes showed a trend towards elevated sENG levels compared to HD exosomes. As previous studies revealed lower plasma sENG levels in HHT patients than in HDs [38,39], our finding suggests that sENG might be preferably packaged into exosomes, where it is protected by the vesicle membrane, instead of being released for free circulation. Further, the correlation between TEP and exosomal sENG levels in HHT patients only suggests a different protein distribution of sENG in HHT patients compared to HDs. The higher the TEP concentration, the less sENG is carried by HHT exosomes. Lower sENG levels could either be the cause of higher TEP concentrations or a result of it. They might lead to a (compensatory) upregulation of other cargo proteins and thus to an increase in TEP. Alternatively, high levels of other cargo proteins might displace sENG levels in the same exosome. Further studies are required to fully understand the results.

Besides the TEP-sENG correlation, another factor distinguishing HHT from HD exosomes was found during the analysis of angiogenesis-related proteins, some of which were previously described as potential HHT biomarkers, but with scarce evidence [40]. Thrombospondin-1 was found at significantly higher and more consistent levels in HHT compared to HD exosomes. Thrombospondin-1 is a matricellular protein involved in the regulation of several cellular activities such as cell proliferation, migration, and apoptosis of different cell types [41]. Moreover, it activates TGF-ß and inhibits angiogenesis. Since the angiogenesis of endothelial cells is disrupted in HHT patients, the higher level of Thrombospondin-1 found in HHT exosomes may indicate the body’s attempt to restabilize the angiogenetic and anti-angiogenetic balance. Several studies have shown the benefits of anti-angiogenetic treatment of HHT models in vitro [15,42]. Therefore, it is plausible that Thrombospondin-1 is increased in HHT exosomes as a possible response to pathologic cellular activities in HHT patients.

Functional assays with HUVECs showed the ability of exosomes to disrupt the cytoskeleton structure and to reduce cell migration and proliferation after internalization by endothelial cells. Since the F-Actin structure is disrupted in HHT endothelial cells due to the F-Actin-ENG interaction [11], a disorganizing effect of HHT exosomes was expected. Migration, proliferation, and angiogenesis are important endothelial functions that are known to be disrupted in HHT endothelial cells as well [10]. The expected effects of HHT exosomes were confirmed. However, similar effects were visible by HD exosomes. 

For both exosomal cargo analysis and functional assays, our study assessed total exosomes, i.e., they represent a mixture of exosomes released from all cell types in the body. The pathologic exosome subset in HHT settings is most likely derived from endothelial cells and their functional effects could be masked by the presence of other exosome subsets. Thus, it can be possible that exosomes derived from HHT endothelial cells have different effects on HUVECs than exosomes released from healthy endothelial cells. Functional experiments with usage of endothelium-derived exosomes will be necessary for a better understanding of HHT pathophysiology. Once the biological mechanisms and role of exosomes in HHT patients are better understood, engineered exosomes [43] could represent a new therapeutic strategy by targeting endothelial cells with altered TGF-ß signaling and revert the imbalance of angiogenic and anti-angiogenic factors.

Moreover, several exosomal characteristics turned out to be possible candidates for the disease severity assessment as they correlated significantly with the clinical severity parameters of HHT patients. As such, exosomal ALK1 may indicate levels of disease severity in terms of the number of consultations and interventions, and exosomal ALK5 might be useful for epistaxis severity assessment. An increase in exosome concentration with increasing ESS suggests a possible stress-induced exosomal release triggered by epistaxis. Thus, the exosomal particle concentration can be useful for epistaxis severity assessment as well.

The study outcomes are mainly limited by the small number of patients. It is challenging to generate big cohort studies as the disease is rare, and blood draw is ethically not reasonable during the acute phase due to the occurring blood loss and already low hemoglobin levels. Additionally, the majority was not genetically tested, which does not allow conclusions to be drawn regarding differences in exosome characteristics dependent on the disease underlying genetic mutation. A variety of genes can lead to the clinical appearance of HHT [44,45], and different genetic mutations can lead to different symptoms. For example, HHT type 1 is more likely associated with lung and brain involvements, and type 2 is more likely associated with liver involvements [46]. Thus, differences in exosomes depending on the different genetic mutations are likely and need to be assessed in a bigger cohort. Another limitation is the usage of total exosomes over the subset of endothelial-derived exosomes, as previously discussed. Due to limited availability of patient material, a further subset analysis of plasma exosomes was not feasible in the frame of this first pilot study.

## 5. Conclusions

This study revealed exosomal Thrombospondin-1 as a potential liquid biomarker for HHT patients. Furthermore, ALK1 revealed to be a potential marker for disease severity in HHT patients, whereas ALK5 and exosomal particle concentration emerged to be potential markers for epistaxis severity. These findings have the potential to improve the HHT diagnostic assessment, which is currently based on clinical criteria in most cases. Not only could liquid biomarkers make the diagnosis happen easier and faster, but also allow a more detailed assessment of disease severity and progression. It is desirable that HHT could be diagnosed with validated biomarkers after just one incident of nose bleeding, while, currently, the diagnostic procedure can take many years depending on the frequency of symptoms or bleedings. Moreover, the serial exosome analysis of HHT patients may give insights into the changes in molecular cargo and be used as a treatment monitoring or disease relapse detection tool as shown by exosome research on head and neck cancer [47].

## Figures and Tables

**Table 1:** Antibodies for Western blot.

Antibody	Supplier, Cat.No.	Dilution	Species	MW	Condition	Membrane
CD63	Invitrogen (Waltham, MA, USA), 10628D	1:1000 in 5% milk	Mouse	30–60 kDa	Non-reducing	Nitrocellulose
CD9	Invitrogen (Waltham, MA, USA), 10626D	1:1000 in 5% milk	Mouse	24 kDa	Non-reducing	Nitrocellulose
TSG101	Invitrogen (Waltham, MA, USA), PA5-31260	1:500 in 5% milk	Rabbit	46 kDa	Reducing	Nitrocellulose/PVDF
ApoA1	Cell Signaling Technology CST (Danvers, MA, USA), 3350	1:1000 in 5% milk	Mouse	25 kDa	Reducing	Nitrocellulose
Grp94	Cell Signaling Technology CST (Danvers, MA, USA) 2104	1:1000 in 5% BSA	Rabbit	100 kDa	Reducing	Nitrocellulose
Soluble Endoglin	Santa Cruz (Santa Cruz, CA, USA), SC-20072	1:200 in 5% BSA	Mouse	61 kDa	Reducing	PVDF

**Table 2:** Clinicopathological data of HHT patients during disease progression and HDs enrolled in this study.

Characteristics	HHT Patients (n = 20)		HD (n = 17)	
	n	%	n	%
Age (years)				
<40	2	10	2	12
40–60	10	50	9	53
>60	8	40	6	35
(range: 22–87)				
Gender				
Female	11	55	9	53
Male	9	45	8	47
HHT manifestation				
Nose	19	95		
Mouth/tongue/throat	12	60		
Lip	9	45		
HEP (liver)	5	25		
PUL (lung)	6	30		
GI (gastrointestinal tract)	3	15		
BRA (brain)	1	5		
ESS [17]				
0–2	1	5		
2–4	5	25		
4–6	10	50		
>6	3	15		
No score	1	5		
Total consultations				
0–9	7	35		
10–24	4	20		
25–49	5	25		
50–99	1	5		
>99	3	15		
Total interventions				
0–9	13	65		
10–24	3	15		
25–49	3	15		
>49	1	5		

ESS = Epistaxis severity score.

## Data Availability

All data relevant to the study are included in the article or uploaded as Supplementary Materials.

## Supplementary Materials

The following supporting information can be downloaded at https://www.mdpi.com/article/10.3390/jcm13185430/s1, Table S1: Curaçao criteria met by individual patients of the studied HHT cohort at the time of their first consultation in our department, on which diagnosis was based; Table S2: Detailed interventions for individual patients of the studied HHT cohort; Figure S1: Migration of HUVECs after exosome incubation.
