# Supplementary material for: Molecular and Functional Cargo of Plasma-Derived Exosomes in Patients with Hereditary Hemorrhagic Telangiectasia
Source: J Clin Med. 2024 Sep 13;13(18):5430. doi: 10.3390/jcm13185430 (PMC11432581; doi:10.3390/jcm13185430)
Supplement: Supplementary file 1 [file jcm-13-05430-s001.zip › jcm-3179472-supplementary.pdf]

Journal of
Clinical Medicine

MDPI

Supplementary Materials for Article

Molecular and functional cargo of plasma-derived exosomes in patients with Hereditary Hemorrhagic Telangiectasia

Table S1: Curaçao criteria met by individual patients of the studied HHT cohort at the time of their first consultation in our department, on which diagnosis was based.

	**Curaçao criteria**				**Number of fulfilled criteria**	**Genetic testing**
**ID**	**Epistaxis**	**Telangiectasia**	**Family history**	**AVM**		
**#1**	x	x	x	-	3	ENG
**#2**	x	x	x	-	3	SMAD4
**#3**	x	x	x	-	3	-
**#4**	x	x	x	PUL	3	-
**#5**	x	x	x	-	4	-
**#6**	x	x	x	-	3	-
**#7**	x	x	x	-	3	-
**#8**	x	x	x	-	3	-
**#9**	x	x	x	-	3	-
**#10**	x	x	unclear	-	2,	-
**#11**	x	x	x	-	3	-
**#12**	x	x	unclear	-	2	-
**#13**	x	x	x	-	3	-
**#14**	x	x	x	HEP	4	-
**#15**	x	x	x	-	3	-
**#16**	x	x	x	HEP	4	-
**#17**	x	x	x	-	3	-
**#18**	x	x	x	-	3	-
**#19**	x	x	x	-	3	-
**#20**	x	x	x	-	3	-

AVM = arterio-venous malformations, PUL = pulmonary, HEP = hepatic

Table S2: Detailed interventions for individual patients of the studied HHT cohort.

	Interventions						
**ID**	**Laser therapy**	**Septal splint**	**Cauterization**	**Complete nasal closure (Young's procedure)**	**Clipping A. sphenopalatina**	**Ligation A. carotis externa, tracheostomy**	**Total**
#1	7 (nose, lips)	-	-	-	-	-	7
#2	12 (nose)	-	-	-	-	-	12
#3	10 (nose, lips)	1	-	-	-	-	11
#4	6 (nose)	-	-	-	-	-	6
#5	2 (nose, face)	2	-	-	-	-	4
#6	47 (nose)	-	1	-	-	-	48
#7	36 (nose, face)	-	3	2	-	-	41
#8	1 (face)	-	-	-	-	-	1
#9	-	-	-	-	-	-	0
#10	16 (nose)	1	-	-	-	-	17
#11	-	1	-	2	-	-	3
#12	-	-	-	-	-	-	0
#13	-	-	-	-	-	-	0
#14	2 (nose, mouth)	2	4	-	1	-	9
#15	57 (nose)	1	-	-	-	-	58
#16	2 (nose)	-	-	-	-	-	2
#17	-	-	-	-	-	-	0
#18	26	4	1	-	1	2	34
#19	-	-	-	-	-	-	0
#20	-	-	-	-	-	-	0
**Total**	**224**	**12**	**9**	**4**	**2**	**2**	**253**

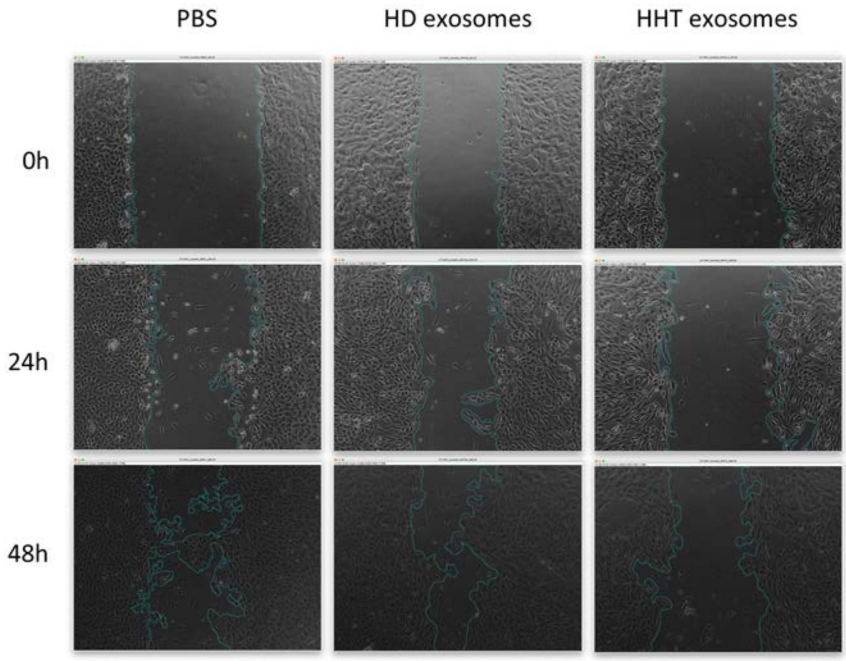

Figure S1: Migration of HUVECs after exosome incubation.
